# Whole-genome analysis reveals the tandem duplication of *Cannabis sativa* L. *GATA* and their stress-responsive expression during seed germination

**DOI:** 10.1186/s12870-026-09308-w

**Published:** 2026-06-29

**Authors:** Elphas Sambu Simiyu, Ye Che, Guo Chao Qi, Zeyu Jiang, Ling Zhang, Wei Yang, Bramwel Waswa Wanjala, Aminu Shehu Abubakar, Gang Gao, Aiguo Zhu, Di Wang

**Affiliations:** 1 Daqing Branch of Heilongjiang Academy of Agricultural Sciences, Daqing, 163319 China; 2 Institute of Bast Fiber Crops, Chinese Academy of Agricultural Sciences, Changsha, 410205 China; 3 Yuelushan Laboratory, Changsha, 410128 China; 4 Kenya Agricultural and Livestock Research Organization, Nairobi, 57811 Kenya; 5 Department of Agronomy, Bayero University Kano, PMB 3011, Kano, Nigeria

**Keywords:** *Cannabis sativa* L., *GATA* transcription factors (TFs), Transcriptome profiling, Abiotic stress tolerance, Fiber crops

## Abstract

**Background:**

GATA transcription factors (TFs) are zinc finger proteins that regulate diverse developmental and stress-responsive processes in plants. Despite the growing economic importance of *Cannabis sativa* L. (hemp) as a source of fiber, seed oil, and bioactive compounds, the GATA TF gene family has not been systematically characterized in this species. This study aimed to perform a comprehensive genome-wide identification and characterization of GATA TF genes in *C. sativa* L. and to investigate their expression responses during early seed germination under abiotic stress conditions.

**Results:**

A total of 17 GATA TF genes (*CsGATAs*) were identified in the *C. sativa* L. genome and phylogenetically classified into three clusters: Cluster I (12 members), Cluster II (one member), and Cluster III (four members). Evidence of localized gene family expansion was observed, with tandem duplication events identified in *CsGATA2*, *CsGATA5*, *CsGATA6*, *CsGATA10*, *CsGATA11*, and *CsGATA14*, distributed across Clusters I and III. Gene structure analysis revealed significant variation, with exon numbers ranging from 2 to 10. Promoter analysis of the 2000 base pairs (bp) upstream regions showed enrichment of stress and hormone-responsive *cis*-acting elements, with abscisic acid (ABA) responsive elements present in 12 of the 17 promoters. Transcriptomic profiling during early germination under cold stress (4 °C), salt stress (200 mM NaCl), and combined cold and salt stress revealed distinct expression patterns. *CsGATA3* was consistently downregulated across all three stress conditions (log_2_FC of -1.14 under salt stress, -1.08 under cold stress, and -1.23 under combined stress). In contrast, *CsGATA9* and *CsGATA14* responded specifically to combined stress, with *CsGATA9* downregulated (log_2_FC of -1.62) and *CsGATA14* upregulated (log_2_FC of + 1.62). qRT-PCR analysis of five differentially expressed *CsGATA* genes confirmed the RNA-seq expression trends, showing strong concordance between the two platforms (Pearson r = 0.83, *p* < 0.001).

**Conclusions:**

This study provides the first genome-wide characterization of the GATA TF family in *C. sativa* L. and reveals gene family expansion driven by tandem duplication. The identification of stress-responsive members, particularly *CsGATA3* as a broadly downregulated gene across all stress conditions and *CsGATA14* as specifically induced under combined stress, highlights candidate transcriptional modulators of abiotic stress adaptation during hemp seed germination. These findings lay a foundation for functional studies and molecular breeding strategies aimed at improving stress tolerance in hemp.

**Supplementary Information:**

The online version contains supplementary material available at https://doi.org/10.1186/s12870-026-09308-w.

## Introduction

Abiotic stresses, including drought, salinity, and extreme temperatures, are major constraints on crop growth and productivity, reducing yields and compromising the structural integrity of fiber tissues [1–5]. In fiber crops such as industrial hemp (*Cannabis sativa* L.), these stresses disrupt secondary cell wall biosynthesis, leading to reduced cellulose deposition, induced lignification, and altered fiber properties [6, 7]. As industrial hemp is increasingly cultivated globally for textiles, composites, and biobased materials [8], elucidating the molecular mechanisms involved in its growth, development, and adaptation to abiotic stresses is crucial for sustaining stable production and fiber quality.

Plants have evolved sophisticated mechanisms to perceive and respond to environmental changes, including reactive oxygen species (ROS) signaling, hormone-mediated pathways, and transcriptional reprogramming that modulate growth, metabolism, and defense [9, 10]. Among transcriptional regulators, the GATA family of zinc finger proteins is associated with responses to environmental and developmental stimuli [11]. These transcription factors (TFs) are evolutionarily conserved across eukaryotes, recognizing the consensus DNA motif (A/T)GATA(A/G) through a single, highly conserved type-IV zinc finger domain (C-X2​-C-X17-20​-C-X2​-C) followed by a basic region. In angiosperms, the GATA family is typically large and diverse, with about 30 members in *Arabidopsis thaliana* and *Oryza sativa* L. [11, 12].

Functionally, plant GATA TFs participate in a wide range of developmental and physiological processes. For example, *A. thaliana GATA21*/*GNC* and *GATA22*/*GNL* regulate the transition from heterotrophic to autotrophic growth by coordinating chloroplast development, greening, and photosynthesis, while integrating hormonal and nitrogen-related signals that influence flowering time and senescence [12, 13]. More broadly, GATA TFs are involved in seed germination, root and leaf development, and responses to abiotic stresses such as cold, drought, and salinity. In *O. sativa* L., *OsGATA16* enhances tolerance to cold stress at the seedling stage, and other members are critical for salt and drought stress responses [14]. Recent genome-wide characterizations in crops such as wheat and grapevine have further highlighted the crucial roles of *GATA* genes in mediating multi-stress resilience [15, 16].

Despite their recognized importance, a systematic characterization of the GATA TF family in *C. sativa* L. has not yet been reported. Key gaps remain regarding their phylogenetic relationships, genomic organization, and regulatory roles under abiotic stress. This is particularly during seed germination, a developmental stage highly sensitive to environmental conditions and critical for stand establishment and subsequent fiber yield [17]. In hemp production systems, early-season germination often coincides with low soil temperatures and residual salinity, making combined cold and salt stress a recurrent agronomic challenge [18, 19]. Moreover, the potential involvement of *C. sativa* L. *GATA* (*CsGATA*) genes in linking environmental stress responses with pathways influencing secondary cell wall formation, a primary determinant of fiber strength and quality, remains largely unexplored.

To address these critical knowledge gaps, this study conducted a comprehensive genome-wide identification and characterization of GATA TFs in *C. sativa* L. Using phylogenetic, conserved motif, synteny, promoter, and transcriptomic analyses under cold, salt, and combined cold-salt stress conditions, we identified candidate *CsGATA* genes potentially involved in abiotic stress-responsive regulatory pathways during early seed germination. In addition, alternative splicing analysis provided novel insights into the structural diversification and post-transcriptional regulation of the *CsGATA* family under stress. Compared with previous *GATA* family studies in model plant species, this study uniquely integrates combined abiotic stress transcriptomics with evolutionary and post-transcriptional alternative splicing analyses in an economically important industrial fiber crop. These findings establish a robust molecular framework for future functional studies and targeted molecular breeding strategies aimed at improving stress resilience, seed vigor, and agronomic performance in hemp.

## Materials and methods

### Plant material, germination assay, and stress treatments

Seeds of *C. sativa* L. var. ‘Qingdama 5’ were obtained from the Gene Bank of the Daqing branch of Heilongjiang Academy of Agriculture Science. ‘Qingdama 5’ is a high-yielding, fiber-oriented cultivar characterized by strong lodging resistance (< 5% under field conditions), an erect stem architecture, and significant tolerance to saline-alkaline soils (pH 7.2–8.5) supported by a well-developed root system. The cultivar has high fiber yield (9,015.3 kg ha^−1^), enhanced resistance to gray mold and stem rot compared with traditional varieties, and completes its growth cycle within 105–115 days. Seeds were stored at 4 °C in sealed foil packets with silica gel desiccant and used within six months of the harvest date. Viability was confirmed prior to the experiment by a preliminary germination test conducted according to ISTA protocols [20]; only seed lots with ≥ 85% germination under control conditions were used.

Seeds were surface sterilized in 2% (v/v) sodium hypochlorite for 10 min with gentle agitation and rinsed five times with sterile distilled water. A 7 day germination assay was conducted to evaluate the effects of cold stress, salt stress, and their combination on seed germination. Fifty seeds per dish were placed in 90 mm Petri dishes lined with two layers of sterile Whatman No. 1 filter paper moistened with 10 mL of sterile distilled water or NaCl solution as specified. To prevent evaporation while allowing gas exchange, dishes were sealed with Parafilm and perforated with three 1 mm pinholes. Moisture levels were checked daily; an additional 2 mL of the corresponding solution (distilled water or NaCl of the same concentration) was added when visual drying of the filter paper was observed. Each treatment consisted of three independent biological replicates, and all dishes were arranged in a completely randomised design within the incubator, with positions rotated 90° daily to minimise positional effects.

Salt concentrations were determined based on preliminary dose–response assays (0–500 mM NaCl) to identify levels that inhibited germination without causing complete lethality (Figure S1). In the preliminary assay, seeds (50 per dish, three replicates per concentration) were germinated in five NaCl concentrations (100, 200, 300, 400, and 500 mM) at 25 ± 1 °C under the standard conditions described above. The 200 mM NaCl concentration was selected for the main experiment as it produced consistent partial inhibition of germination without complete lethality.

Four treatments were applied: (i) control, seeds imbibed in distilled water (0 mM NaCl) at 25 ± 1 °C; (ii) cold stress, seeds exposed to 4 °C for 12 h in darkness during early imbibition and then transferred to 25 ± 1 °C; (iii) salt stress, seeds incubated in 200 mM NaCl at 25 ± 1 °C for 72 h followed by transfer to distilled water for recovery; and (iv) combined stress, seeds exposed to 200 mM NaCl at 4 °C for 12 h, then maintained in 200 mM NaCl at 25 ± 1 °C until 72 h and subsequently transferred to distilled water. Following any cold treatment, all dishes were maintained at 25 ± 1 °C under a 16/8 h light/dark photoperiod with a photosynthetic photon flux density of 120 μmol m^−2^ s^−1^. All 25 °C incubations were conducted in a programmable illuminated growth chamber. Cold treatments (4 °C) were performed in a separate temperature-controlled incubator; temperature accuracy was verified daily using a calibrated digital thermometer placed at dish level. NaCl solutions were prepared using analytical-grade NaCl dissolved in sterile distilled water.

Germination was recorded daily for 7 days, and seeds were considered germinated upon radicle emergence ≥ 2 mm. Counts were performed at the same time each day (09:00 h). Seeds showing visible fungal contamination were removed and recorded separately; replicates in which contamination exceeded 5% of the total seeds per dish were excluded from analysis. Germination percentage (GP) and mean germination time (MGT) were calculated according to Ellis and Roberts [21]:

$$GP \left(\%\right) =(\frac{n}{N})\times 100$$

$$MGT = \frac{\sum (ni \times di)}{\sum ni} =\frac{\sum (ni \times di)}{n}$$

where *n* is the total number of germinated seeds, *N* is the total number of seeds tested, *n*_*i*_ is the number of seeds germinated on day *d*_*i*_, and *d*_*i*_ is the number of days from the start of imbibition.

The germination index (GI) [22] was calculated as:

$$GI= \sum \frac{Gi}{di}$$

where G_i_ is the number of seeds germinating on day d_i_.

Time to 50% germination (T₅₀) was derived from a three-parameter logistic model fitted to cumulative germination curves:

$$G(t)=\frac{{G}_{max}}{1+\mathrm{e}\mathrm{x}\mathrm{p}\left(-k\left(t-{T}_{50}\right)\right)}$$

where G(t) is cumulative germination percentage at time t, G_max_ is the estimated maximum germination percentage, k is the rate constant, and T_50_ is the time at which 50% of G_max_ is reached. Model fitting was performed using the *drc* package in R [23].

All germination parameters (GP, MGT, GI, and T_50_) were subjected to one-way analysis of variance (ANOVA) followed by Tukey’s Honest Significant Difference (HSD) post-hoc test for pairwise comparisons among treatments. Homogeneity of variance was verified using Levene’s test prior to ANOVA. Where assumptions of normality or homoscedasticity were violated, a non-parametric Kruskal–Wallis test was applied as a confirmatory analysis, with Dunn’s post-hoc test used for multiple comparisons. All statistical analyses were performed in R (v4.5) using the *agricolae*, *stats*, and *FSA* packages. Significance was defined at *p* < 0.05. All data are presented as mean ± standard error (SE) of three independent biological replicates.

For gene expression analyses, seedlings were grown under identical conditions and harvested at 48 h post-imbibition, corresponding to early germination prior to recovery in salt treatments. At harvest, the entire seedling (radicle and hypocotyl) was collected. For each of three biological replicates, 20 seedlings were pooled, flash-frozen in liquid nitrogen within 60 s of collection, and stored at −80 °C to capture early stress-responsive transcriptional events.

### Identification and sequence analysis of *CsGATA* genes

The *C. sativa* L. reference genome (CS10, National Center for Biotechnology Information (NCBI BioProject PRJNA557030)) was queried using HMMER (v3.3.2) [24] with the ZnF_GATA zinc finger profile (Pfam: PF00320) [25] (E-value < 1 × 10^–5^). Candidate sequences were retained only if confirmed by at least one additional database (SMART [26] or NCBI CDD [27]). Sequences lacking the conserved zinc-finger motif (C-X_2_-C-X_17–20_-C-X_2_-C) upon manual inspection of the multiple sequence alignment were excluded. Predicted gene models were cross-validated against the CS10 genome annotation (annotation release 100) to confirm exon–intron boundaries and coding sequence (CDS) integrity, and genes with incomplete open reading frames (ORF) or annotation conflicts were excluded from downstream analyses.

### Phylogenetic analysis

Full-length GATA proteins from *C. sativa* L., *A. thaliana* (TAIR10), and *O. sativa* L. (IRGSP-1.0) were aligned with MAFFT (v7.505) [28] using the L-INS-i algorithm. Poorly aligned regions were trimmed with trimAl (v1.4) [29]. Maximum likelihood (ML) phylogeny was inferred using IQ-TREE (v2.3) [30] with ModelFinder [31] selected substitution models, 1000 ultrafast bootstrap replicates [32], and 1000 SH-aLRT tests. Trees were visualized and annotated using iTOL [33].

### Chromosomal mapping, synteny, and motif analyses

Chromosomal locations were obtained from CS10 genome annotations and visualized with TBtools-II (v2.329) [34, 35]. Synteny was assessed with MCScanX [36] using an E-value threshold of 1 × 10^–5^ and a minimum block size of five genes. Conserved motifs were predicted with Multiple Em for Motif Elicitation (MEME Suite—v5.5.5) [37] using a maximum of 10 motifs, motif width of 6–50, and E-value < 1 × 10^–5^. Promoter sequences (2000 base pairs (bp) upstream of each start codon) were analyzed with PlantCARE [38] for hormone and stress-responsive *cis*-elements.

### Physicochemical property and subcellular localization analysis

The physicochemical properties of CsGATA proteins, including amino acid (aa) length, molecular weight (kDa), theoretical isoelectric point (pI), instability index, and grand average of hydropathicity (GRAVY), were calculated using the ExPASy ProtParam tool [39]. Subcellular localization of CsGATA proteins was predicted using WoLF PSORT [40] with default parameters, supplemented by plant-specific localization prediction tools.

### Alternative splicing analysis

Isoform diversity was evaluated using StringTie assemblies [41]. Isoforms with transcripts per million (TPM) > 1 in at least one treatment were retained for downstream analysis. Exon and intron structures were visualized using TBtools-II [35], and plots summarizing alternative splicing patterns were generated using Python.

### RNA extraction and sequencing

Total RNA was extracted from germinating seedlings using the SteadyPure Plant RNA Extraction Kit (Accurate Biotechnology, Changsha, China). RNA integrity was assessed using an Agilent 2100 Bioanalyzer, and only samples with an RNA Integrity Number (RIN) ≥ 7.0 were retained for library construction. RNA concentration and purity were determined using a NanoDrop 2000 spectrophotometer (Thermo Fisher Scientific, USA), with A_260_/A_280_ ratios between 1.8 and 2.1 accepted.

For each sample, 1 μg of high-quality total RNA was used for strand-specific library construction using the NEBNext Ultra II Directional RNA Library Prep Kit for Illumina (New England Biolabs, USA). Poly(A)^+^ mRNA was enriched using oligo(dT) magnetic beads and fragmented into short fragments prior to first-strand cDNA synthesis using random hexamer primers. After second-strand synthesis, end repair, A-tailing, adaptor ligation, and PCR amplification were performed according to the manufacturer’s instructions. The resulting libraries were quantified by qPCR and quality-assessed on an Agilent 2100 Bioanalyzer prior to sequencing.

Libraries were sequenced on an Illumina NovaSeq 6000 platform (Illumina, USA) in paired-end 150 bp mode. An average of approximately 41 million clean reads were obtained per sample (range: 37.8–46.1 million), with Q30 scores exceeding 96% across all libraries (Supplementary Table S2).

### Transcriptome assembly and differential expression analysis

Raw sequencing reads were quality-filtered using fastp (v0.23.1) [42] with the following parameters: adapter trimming enabled, bases with Phred quality scores below 20 removed by sliding window (window size 4 bp), reads with more than five ambiguous (N) bases discarded, and reads shorter than 100 nucleotide (nt) after trimming excluded together with their paired reads. Clean reads were aligned to the *C. sativa* L. reference genome (CS10; NCBI BioProject PRJNA557030, annotation release 100) using HISAT2 (v2.1.0) [43] in strand-specific mode, guided by the CS10 GFF3 annotation file. Alignment rates exceeded 91% for all libraries (Supplementary Table S3).

Transcript assembly and expression quantification were performed using StringTie (v1.3.3b) [41, 44] in reference-guided mode (–rf flag), retaining only annotated gene loci; novel intergenic transcripts were excluded. Gene expression levels were calculated as transcripts per million (TPM) and raw read counts. Genes with total read counts below 10 across all samples were excluded prior to statistical analysis. Differential expression analysis was performed using DESeq2 (v1.12.4) [45] in R. Genes with |log_2_FC|≥ 1 and Benjamini–Hochberg-adjusted *p*-value (q-value) < 0.05 were considered significantly differentially expressed. The following pairwise comparisons were performed: (i) salt stress alone (200 mM NaCl, 25 °C) vs. control (0 mM NaCl, 25 °C); (ii) cold stress alone vs. control; and (iii) combined stress vs. control. Gene functional annotation was based on the CS10 official annotation supplemented by BLASTp searches (E-value < 1 × 10⁻^5^) against the NCBI non-redundant protein database and cross-referenced with UniProt/Swiss-Prot. Heatmaps and expression visualizations were generated using the *pheatmap* and *ggplot2* packages in R.

Co-expression networks were constructed using Pearson correlation coefficients calculated across condition means. An adjacency matrix was built using a correlation threshold of r ≥ 0.75. The resulting network was analyzed using the *igraph* [46] package in R to calculate degree centrality, betweenness centrality, and average correlation strength for hub gene identification.

### qRT-PCR validation and statistical analyses

Total RNA was extracted from the same seedling samples used for RNA-seq using the SteadyPure Plant RNA Extraction Kit (Accurate Biology, Beijing, China). RNA concentration and purity were verified using a NanoDrop 2000 spectrophotometer (Thermo Fisher Scientific, USA), accepting only samples with A_260_/A_280_ ratios between 1.8 and 2.1. RNA integrity was confirmed by 1% agarose gel electrophoresis.

First-strand cDNA was synthesized from 200 ng total RNA using the *Evo M-MLV* RT Mix Kit with gDNA Clean for qPCR Ver.2 (Accurate Biology, Beijing, China) according to the manufacturer’s instructions, which includes an on-column genomic DNA elimination step prior to reverse transcription. Gene-specific primers for five *CsGATA* genes selected for validation (*CsGATA3*, *CsGATA8*, *CsGATA9*, *CsGATA10*, and *CsGATA14*) were designed using Primer3 [47] and are provided in Supplementary Table S4. qRT-PCR was performed on a CFX96™ Real-Time PCR System (Bio-Rad, USA) using SYBR Green chemistry. Thermal cycling conditions were as follows: initial denaturation at 95 °C for 3 min; 40 cycles of 95 °C for 15 s, 60 °C for 30 s, and 72 °C for 30 s; followed by melt curve analysis from 65 °C to 95 °C in 0.5 °C increments to confirm amplicon specificity. *CsActin* was used as the internal reference gene [48]. Relative expression levels were calculated using the 2^-∆∆ct^ method [49], with the 0 mM NaCl control treatment as the calibrator.

All statistical analyses were performed in R (v4.5.0). For germination parameters (GP, MGT, GI, and T₅₀) and qRT-PCR expression data, normality of residuals was assessed using the Shapiro–Wilk test and homogeneity of variance was verified using Levene’s test (R package *car*). Data meeting parametric assumptions were analyzed by one-way ANOVA followed by Tukey’s HSD post-hoc test using the *agricolae* package. Where assumptions were violated, the non-parametric Kruskal–Wallis test with Dunn’s post-hoc test (Benjamini–Hochberg correction; R package *FSA*) was applied as a confirmatory analysis. Agreement between RNA-seq and qRT-PCR expression profiles was assessed by Pearson correlation between |log_2_FC| values from both platforms using the *cor.test()* function, reported at both the overall (all five genes) and per-gene level. Differences were considered statistically significant at *p* < 0.05. All data are presented as mean ± SE of three independent biological replicates.

## Results

### Identification and chromosomal mapping of *CsGATA* genes in *C. sativa* L.

Seventeen *GATA* genes were identified in the *C. sativa* L. reference genome (CS10) and designated *CsGATA1* to *CsGATA17* following the sequential nomenclature conventions established in genome-wide GATA TF characterization studies in other plant species [50–52]. These genes encoded proteins ranging from 151 aa (*CsGATA16*) to 538 aa (*CsGATA8*), with predicted molecular weights between 16.2 and 59.4 kDa and theoretical pI values from 4.75 to 9.89 (Table 1). All GATA proteins exhibited negative GRAVY values (range: −1.025 to −0.542; mean −0.75), indicating predominantly hydrophilic character. Instability indices ranged from 34.85 to 65.41 (mean 51.02), with most values exceeding 40, suggesting variable in vitro stability among family members. Subcellular localization prediction indicated nuclear localization for all 17 *CsGATA* genes, consistent with their role as TFs.

**Table 1:** Physicochemical characteristics, predicted subcellular localization, and chromosomal location of GATA TFs identified in the genome of *C. sativa* L

Gene	Protein ID	Length	MW (kDa)	pI	GRAVY	Instability index	Localization	Chromosome
*CsGATA1*	XP_030480651.1	301	32.46	6.21	−0.654	38.01	Nucleus	NC_044370.1
*CsGATA2*	XP_030480667.1	365	40.40	5.09	−0.679	46.96	Nucleus	NC_044370.1
*CsGATA3*	XP_030487548.1	339	37.16	6.06	−0.721	44.15	Nucleus	NC_044371.1
*CsGATA4*	XP_030487739.1	369	41.33	9.04	−0.973	46.38	Nucleus	NC_044371.1
*CsGATA5*	XP_030488344.1	347	37.72	6.46	−0.566	58.72	Nucleus	NC_044371.1
*CsGATA6*	XP_030498922.1	333	36.57	5.24	−0.777	62.72	Nucleus	NC_044373.1
*CsGATA7*	XP_030499018.1	304	34.12	8.02	−1.025	55.6	Nucleus	NC_044373.1
*CsGATA8*	XP_030501038.1	538	59.42	6.4	−0.648	55.68	Nucleus	NC_044374.1
*CsGATA9*	XP_030502249.1	278	30.99	6.18	−0.867	54.5	Nucleus	NC_044374.1
*CsGATA10*	XP_030503179.1	388	42.16	4.75	−0.542	49.51	Nucleus	NC_044375.1
*CsGATA11*	XP_030504713.1	317	33.89	5.57	−0.656	34.85	Nucleus	NC_044375.1
*CsGATA12*	XP_030505639.1	181	19.65	9.89	−0.75	40.56	Nucleus	NC_044375.1
*CsGATA13*	XP_030506133.1	325	36.15	9.1	−0.765	49.72	Nucleus	NC_044375.1
*CsGATA14*	XP_030508431.1	267	30.29	8.69	−0.777	54.21	Nucleus	NC_044376.1
*CsGATA15*	XP_030508436.1	273	30.37	9.17	−0.697	60.44	Nucleus	NC_044376.1
*CsGATA16*	XP_030508484.1	151	16.17	9.88	−0.948	65.41	Nucleus	NC_044376.1
*CsGATA17*	XP_030511114.1	352	38.28	4.98	−0.714	49.91	Nucleus	NC_044377.1

**Fig. 1:**
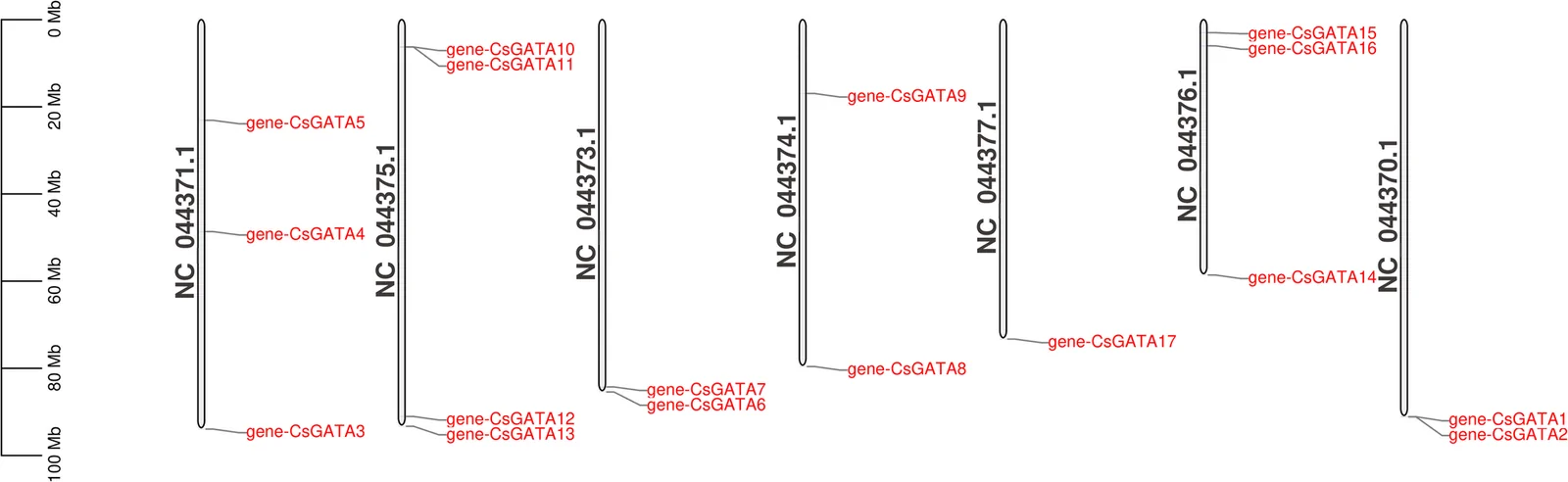
Chromosomal location and distribution of *CsGATA* genes in *C. sativa* L. Seventeen identified *CsGATA* genes were mapped onto the ten assembled chromosomes of the *C. sativa* L. reference genome (CS10). The genes were unevenly distributed across seven chromosomes, with no genes found on NC_044372.1, NC_044378.1, or NC_044379.1

Chromosomal mapping showed that *CsGATA* genes were unevenly distributed across seven of the ten assembled chromosomes, with no members detected on NC_044372.1, NC_044378.1, or NC_044379.1 (Fig. 1). NC_044375.1 harbored the highest number of *CsGATA* genes (four), followed by NC_044371.1 and NC_044376.1 with three genes each. Two genes were located on NC_044370.1, NC_044373.1, and NC_044374.1, while NC_044377.1 contained a single member (*CsGATA17*). The physical clustering of *CsGATA10*/*CsGATA11* and *CsGATA14*-*CsGATA16* suggests that tandem duplication events may have contributed to the expansion of this gene family in *C. sativa* L.

### Gene structure and conserved motifs of CsGATA TFs

**Fig. 2:**
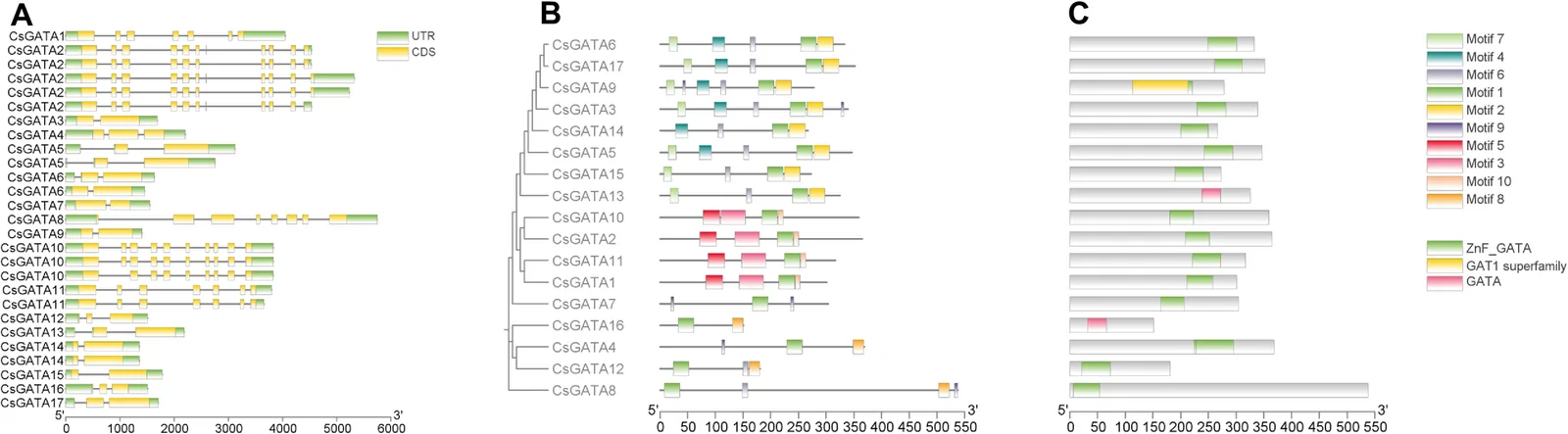
Gene structure and conserved motifs of CsGATA TFs. **A** Exon–intron structures of 17 *CsGATA* genes. Gene lengths are drawn to scale. **B** Conserved motifs in CsGATA proteins identified by MEME analysis. **C** Conserved domain architecture of CsGATA proteins predicted by SMART and CDD, showing the ZnF_GATA domain

The 17 *C. sativa* L. *GATA* genes showed significant diversity in exon and intron organization (Fig. 2A). Exon numbers ranged from 2 to 10, with corresponding intron counts from 1 to 9. *CsGATA2* and *CsGATA10* were the most structurally complex, each comprising 10 exons and 9 introns, whereas *CsGATA3*, *CsGATA7*, *CsGATA9*, *CsGATA14*, and *CsGATA15* had only 2 exons and a single intron. Most other genes, such as *CsGATA4-6*, *CsGATA12-13*, and *CsGATA16-17*, had an intermediate structure of 3 exons and 2 introns.

Domain analysis confirmed the universal presence of the ZnF_GATA zinc finger across all *CsGATA* genes (Supplementary Table S5). Most members aligned with established domain models such as SMART ZnF_GATA (smart00401) or CDD ZnF_GATA (cd00202). However, *CsGATA9* showed a weaker association with the GAT1 superfamily, suggesting possible divergence from canonical GATA functions. In addition, de novo motif discovery with MEME identified 10 statistically significant motifs across the family (E-value < 0.05; Fig. 2B). Motif 1, which corresponded precisely to the canonical GATA zinc finger, was detected in all 17 members with high conservation. Motif 2 was observed in a subset of longer proteins, positioned immediately C-terminal to the zinc finger. Motif 3, observed only in *CsGATA1*, *CsGATA2*, *CsGATA10*, and *CsGATA11*, was strongly enriched in basic residues, a pattern characteristic of nuclear localization signals. Several other accessory motifs were distributed unevenly across the family.

The overall distribution of motifs was consistent with phylogenetic clustering. Genes belonging to the *CsGATA1/2/10/11* cluster shared a common suite of accessory motifs. In contrast, those with simplified exon and intron structures, such as *CsGATA14* and *CsGATA15*, retained only the core zinc finger and a minimal set of additional elements. Intron-rich members, such as *CsGATA2* and *CsGATA10*, carried the most diverse motif repertoires, indicating that structural complexity and motif diversification tend to coincide within the GATA family.

### Phylogenetic analysis of GATA TFs in *C. sativa* L.

**Fig. 3:**
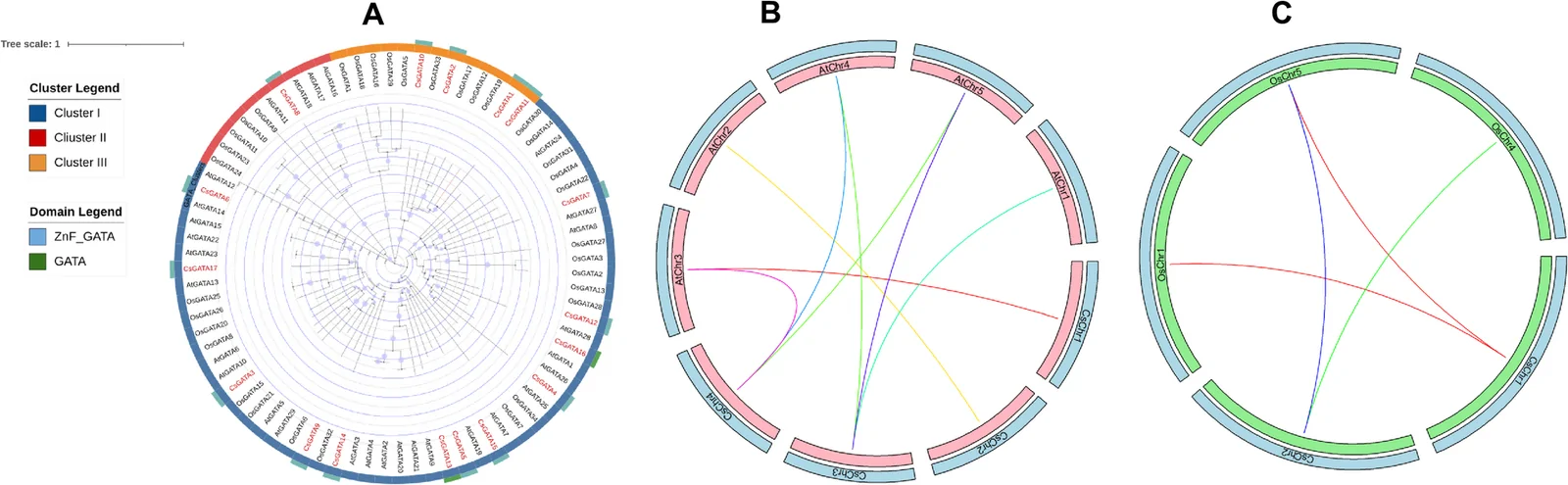
Phylogenetic relationships and synteny analysis of GATA TFs. **A** ML phylogenetic tree constructed using full-length GATA protein sequences from *C. sativa* L., *A. thaliana*, and *O. sativa* L. **B** Synteny relationships between *C. sativa* L. and *A. thaliana GATA* genes. **C** Synteny relationships between *C. sativa* L. and *O. sativa* L. *GATA* genes. Colored lines indicate conserved collinear gene pairs between species

Phylogenetic analysis of the GATA TF family across *C. sativa* L., *O. sativa* L., and *A. thaliana* resolved the sequences into three well supported clusters (Fig. 3A). In *C. sativa* L., all 17 *GATA* genes were distributed among these clusters, with Cluster I having 12 members, including *CsGATA3*, *CsGATA4*, *CsGATA5*, *CsGATA6*, *CsGATA7*, *CsGATA9*, *CsGATA12*, *CsGATA13*, *CsGATA14*, *CsGATA15*, *CsGATA16*, and *CsGATA17*. This cluster represented the largest group. Cluster II had a single gene, *CsGATA8*. Cluster III had four members: *CsGATA1*, *CsGATA2*, *CsGATA10*, and *CsGATA11*. Notably, Cluster I members were dominated by conserved ZnF_GATA and GATA domains, suggesting functional conservation within this subgroup. Cluster III members, although fewer, included both *O. sativa* L. and *C. sativa* L. *GATA* genes, indicating potential evolutionary divergence between monocot and dicot lineages. Cluster II only had *CsGATA8*, suggesting a divergent lineage, consistent with its unique phylogenetic position.

### Synteny relationships of *GATA* genes

#### *A. thaliana* vs *C. sativa* L.

Synteny analysis between *C. sativa* L. and *A. thaliana* identified conserved collinearity for seven out of the 17 *CsGATA* genes (Fig. 3B). In total, 11 syntenic links were detected, involving *CsGATA5*, *CsGATA6*, *CsGATA7*, *CsGATA12*, *CsGATA13*, *CsGATA15*, and *CsGATA16*. These genes were located on CsChr2, CsChr3, CsChr4, CsChr5, and CsChr6, and corresponded to *Arabidopsis* orthologs distributed across AtChr1, AtChr2, AtChr3, AtChr4, and AtChr5. Notably, *CsGATA6* (CsChr4) and *CsGATA16* (CsChr6) showed multiple syntenic connections with *Arabidopsis* genes, reflecting possible ancient duplication and subsequent retention events. In contrast, single copy syntenic relationships were observed for *CsGATA5* (*At3G54810*, AtChr3), *CsGATA7* (*At2G18380*, AtChr2), *CsGATA12* (*At5G26930*, AtChr5), *CsGATA13* (*At1G08000*, AtChr1), and *CsGATA15* (*At3G24050*, AtChr3). The recurrent mapping of *CsGATA16* to both *At3G06740* (AtChr3) and *At4G16141* (AtChr4) further suggests functional diversification following duplication. Interestingly, 10 *CsGATA* genes lacked detectable synteny with *Arabidopsis*.

#### *O. sativa *L. vs *C. sativa* L.

Comparative synteny analysis revealed that a subset of *C. sativa* L. GATA TFs established conserved genomic blocks with *O. sativa* L. Four syntenic relationships were identified, involving *CsGATA3*, *CsGATA6*, and *CsGATA7* (Fig. 3C). *CsGATA3* had two distinct syntenic links, one with *Oryza* chromosome 1 (*Os01t0745700-01*) and another with chromosome 5 (*Os05t0520300-00*), underscoring its potential evolutionary conservation across multiple genomic regions. *CsGATA6* showed a conserved collinear block with *Os04t0539500-01* on *O. sativa* L. chromosome 4, while *CsGATA7* was syntenic with *Os05t0578900-01* on chromosome 5. No other *C. sativa* L. *GATA* members displayed syntenic relationships with *O. sativa* L. in the present analysis.

### Structural and regulatory diversity of *CsGATA* genes

#### Alternative splicing diversity of *CsGATA* genes

**Fig. 4:**
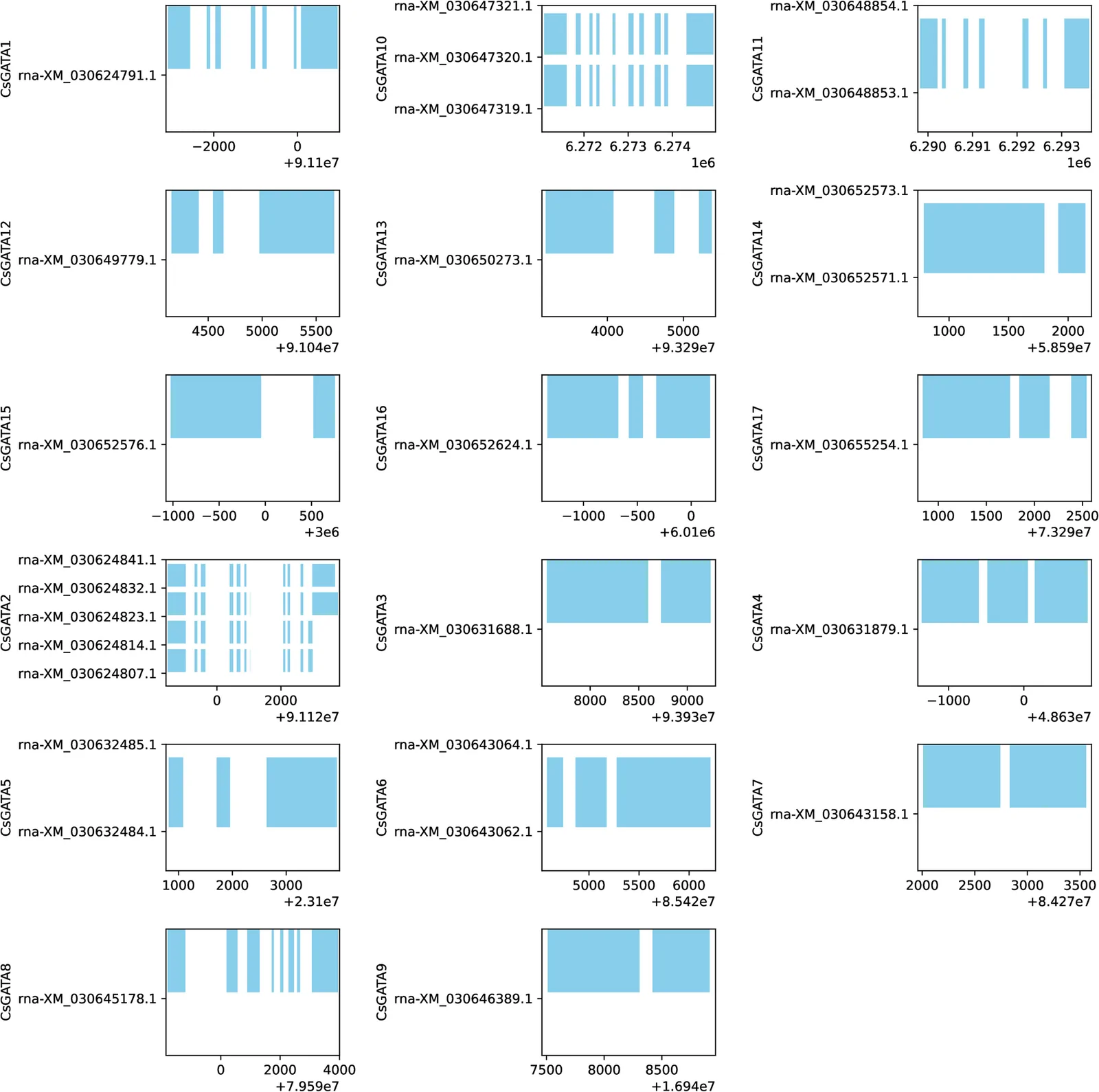
Alternative splicing (AS) patterns and exon–intron organization across the 17 *CsGATA* genes in *C. sativa* L

The 17 *CsGATA* genes showed significant complexity, generating one to five isoforms per gene (Fig. 4). *CsGATA2* had the highest splicing diversity with five transcript variants, having 10–12 exons each. In contrast, *CsGATA13* and *CsGATA16* maintained a single isoform with 3–4 exons, suggesting conserved functional roles. *CsGATA10* had four transcripts with exon variations concentrated at the 5’ and 3’ ends.

*CsGATA1* isoforms demonstrated extended regions of exons in the central CDS, indicating potential modulation of protein-DNA interaction domains. At the isoform level, exon counts per transcript ranged from three (*CsGATA16*) to 12 (*CsGATA2*), with alternative splicing primarily involving exon skipping, observed in nine genes, and alternative donor/acceptor sites (six genes), reflecting gene-specific diversification mechanisms.

#### *Cis*-regulatory element (CRE) landscape of CsGATA promoters

**Fig. 5:**
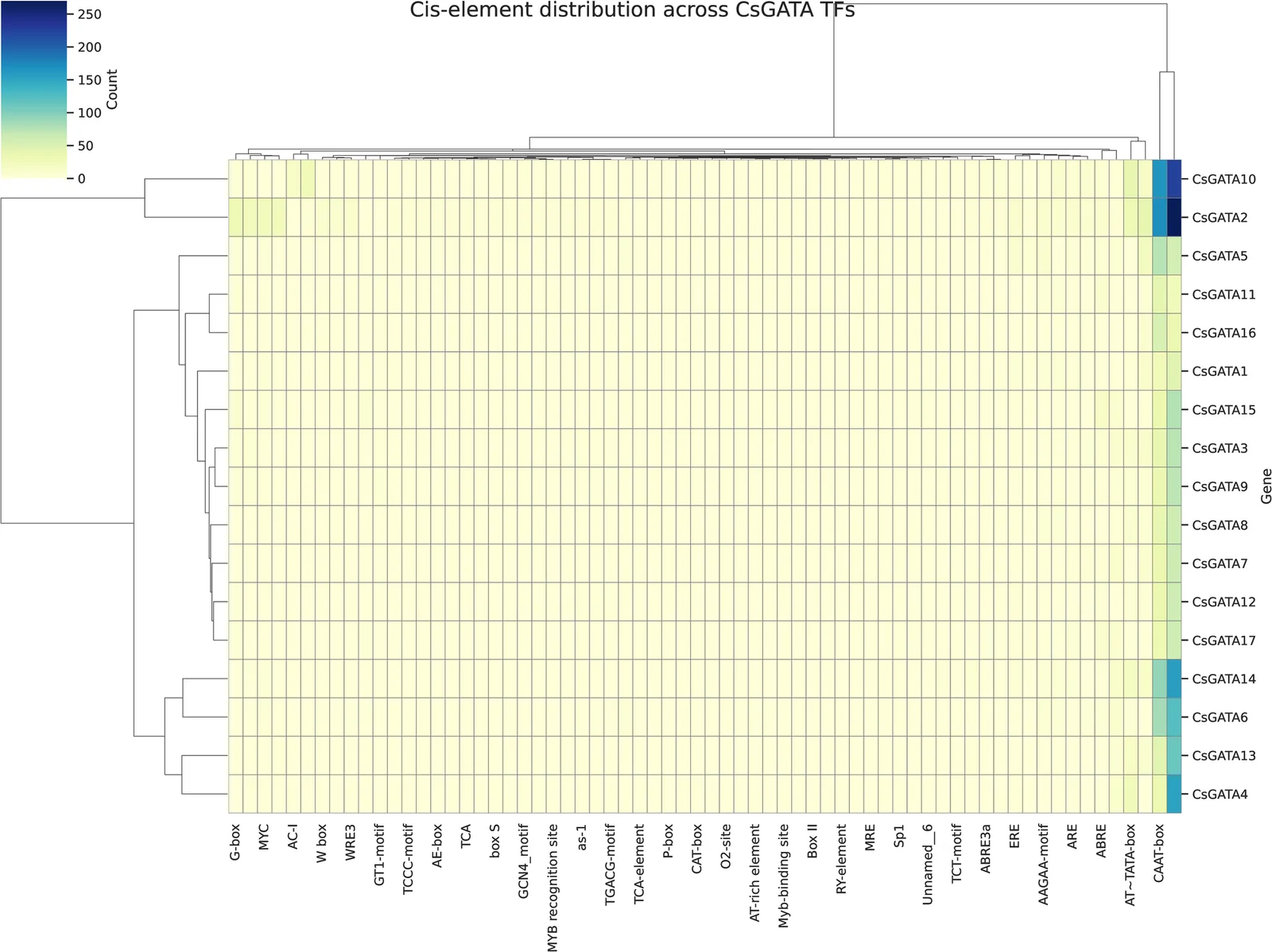
Analysis of CREs within the promoter regions (2000 bp upstream of the ATG start codon) of the 17 *CsGATA* genes, revealing diverse regulatory potential. ABA-responsive elements (ABREs) were the most prevalent

The promoter regions, defined as 2000 bp upstream of the ATG start codon, of the *CsGATA* gene family were analyzed to elucidate the CRE landscape, revealing a highly heterogeneous distribution of motifs associated with phytohormone signaling and environmental response (Fig. 5). Elements responsive to key phytohormones were highly prevalent across the family, implicating the *CsGATA* genes in diverse developmental and stress-response pathways. ABA responsive elements (ABREs) were the most widespread, identified in the promoters of 12 out of 17 *CsGATA* genes, with a frequency ranging from 2–5 motifs per promoter. Genes with notable ABRE abundance included *CsGATA1*, *CsGATA2*, *CsGATA5*, and *CsGATA10*. Furthermore, auxin-responsive TGA-elements were concentrated in the promoters of *CsGATA2*, *CsGATA8*, and *CsGATA11*, suggesting regulatory links to cell elongation and tropisms. Gibberellin-responsive GARE-motifs, typically linked to germination and flowering, were detected in *CsGATA2* and *CsGATA11*. The convergence of multiple hormone-related motifs in the *CsGATA2* promoter indicates its potential function as a key transcriptional hub integrating complex upstream signals.

Regulation via light and stress stimuli was confirmed by the abundance of relevant CREs. Light responsive elements (G-box, Box 4, and AE-box) were found present in 15 of the 17 gene promoters. The highest motif density was observed in the *CsGATA5* and *CsGATA8* promoters, each housing up to six light-related elements, underscoring their potential role in controlling photomorphogenesis and photosynthesis in *C. sativa* L. Stress-specific CREs were also enriched in certain members, conferring enhanced environmental adaptability. Motifs associated with drought response (MBS) and low-temperature (LTR) were significantly clustered in *CsGATA2*, *CsGATA10*, and *CsGATA14*. This specific enrichment strongly suggests that these gene members may play a role as candidates for functional studies focusing on improving abiotic stress tolerance and resilience in *C. sativa* L. cultivars, which is a critical trait for industrial crop productivity.

### Germination performance of *C. sativa* L. var. Qingdama 5 under cold, salt, and combined stress

Germination performance of *C. sativa* L. var. ‘Qingdama 5’ was significantly affected by stress treatment. One-way ANOVA across all 12 treatment groups (four stress regimes × five NaCl concentrations plus control and cold stress alone) revealed a highly significant treatment effect on final germination percentage at day 7 (*p* < 0.001). Levene’s test confirmed homogeneity of variance prior to ANOVA (*p* = 0.500). This result was consistent with Kruskal–Wallis test (*p* = 0.003), validating the parametric analysis.

For the four main treatment groups (control, cold stress, 200 mM salt stress, and combined cold-salt stress at 200 mM NaCl), GP differed significantly (*p* < 0.05; Table 2). The control achieved the highest GP (94.7 ± 2.9%), which was significantly greater than all stress treatments (Tukey’s HSD; *p* ≤ 0.01 for all comparisons with control). Cold stress alone (4 °C, 0 mM NaCl) reduced GP to 48.7 ± 11.2%, though this decline did not differ significantly from salt stress at 200 mM (41.3 ± 6.4%; *p* = 0.895) or combined stress (28.7 ± 6.8%; *p* = 0.298). Combined cold and salt stress produced the lowest GP, though it did not differ significantly from salt stress alone at the same concentration (*p* = 0.640).

**Table 2:** Germination parameters of *C. sativa* L. var. ‘Qingdama 5’ under four main treatments (mean ± SE, *n* = 3)

Treatment	GP (%)	MGT (days)	GI	T_50_ (days)
Control	94.7 ± 2.9 *a*	3.23 ± 0.46 *a*	22.40 ± 3.17 *a*	2.73 ± 0.53
Cold	48.7 ± 11.2 *b*	2.94 ± 0.17 *a*	10.89 ± 2.90 *b*	2.35 ± 0.23
Salt 200 mM	41.3 ± 6.4 *b*	3.95 ± 0.12 *a*	6.05 ± 1.02 *b*	3.43 ± 0.03
Combined	28.7 ± 6.8 *b*	4.20 ± 0.25 *a*	3.69 ± 0.59 *b*	3.60 ± 0.13

MGT showed a significant overall treatment effect (*p* < 0.05), although no individual pairwise comparison reached significance after Tukey’s HSD correction (all *p* > 0.05). MGT was shortest in the cold stress treatment (2.94 ± 0.17 days) and longest under combined stress (4.20 ± 0.25 days; Table 2). The Kruskal–Wallis test returned a non-significant result for MGT (*p* = 0.092), consistent with the Tukey outcome.

The GI was significantly affected by treatment (*p* < 0.05). The control exhibited the highest GI (22.40 ± 3.17), significantly exceeding cold stress (10.89 ± 2.90;* p* < 0.05), salt stress at 200 mM (6.05 ± 1.02; *p* < 0.05), and combined stress (3.69 ± 0.59; *p* < 0.05). No significant differences in GI were detected among the three stress treatments (all *p* > 0.18).

The time to 50% germination (T_50_), derived from three-parameter logistic model fitting to cumulative germination curves (R^2^ > 0.97 for all fits), showed a trend toward later onset under stress conditions. T_50_ was shortest under cold stress (2.35 ± 0.23 days) and control (2.73 ± 0.53 days), and longest under combined stress (3.60 ± 0.13 days) and salt stress (3.43 ± 0.03 days; Table 2). However, neither ANOVA (*p* > 0.05) nor Kruskal–Wallis (*p* > 0.05) reached significance for T_50_ after Tukey correction, and no individual pairwise comparison was significant (all *p* > 0.05).

Across the full dose–response range (100–500 mM NaCl), a clear concentration-dependent reduction in GP was observed under both salt stress alone and combined cold-salt stress (Table 3). Under salt stress, GP declined from 56.0 ± 14.7% at 100 mM to 13.3 ± 2.9% at 500 mM NaCl, accompanied by a progressive increase in MGT from 3.74 ± 0.11 to 5.94 ± 0.24 days. Combined cold-salt stress consistently produced lower GP and higher MGT than the corresponding salt-alone concentration at 200–500 mM NaCl, with the 500 mM combined treatment yielding GP of only 14.0 ± 4.2% and MGT of 6.23 ± 0.34 days.

**Table 3:** Dose-dependent effects of NaCl concentration on germination parameters under salt stress alone (25 °C) and combined cold-salt stress (4 °C), mean ± SE, *n* = 3

NaCl (mM)	GP—Salt (%)	MGT—Salt (d)	GP—Combined (%)	MGT—Combined (d)
100	56.0 ± 14.7	3.74 ± 0.11	39.3 ± 8.1	3.44 ± 0.13
200	41.3 ± 6.4	3.95 ± 0.12	28.7 ± 6.8	4.20 ± 0.25
300	40.0 ± 6.1	5.21 ± 0.27	29.3 ± 2.4	4.53 ± 0.38
400	20.0 ± 2.0	5.15 ± 0.10	18.7 ± 5.5	5.23 ± 0.04
500	13.3 ± 2.9	5.94 ± 0.24	14.0 ± 4.2	6.23 ± 0.34

### Transcriptional expression patterns of *GATA* genes in *C. sativa* L. under abiotic stress

To comprehensively characterize the transcriptional behavior of the *CsGATA* gene family under abiotic stress, expression profiles of all identified *CsGATA* genes were analyzed across salt, cold, and combined stress conditions during germination (Supplementary Table S6). Using stringent differential expression criteria (|log_2_FC|≥ 1 and q < 0.05), only a subset of genes exhibited significant transcriptional responses, whereas the majority showed relatively stable expression patterns with modest fluctuations. Differential expression analyses were mainly interpreted relative to the control treatment (0 mM NaCl, 25 °C) to identify stress-responsive *CsGATA* genes. Additional pairwise comparisons between combined stress and the corresponding single-stress treatments (combined vs. salt and combined vs. cold) were performed to identify transcriptional responses specifically associated with stress interactions.

**Fig. 6:**
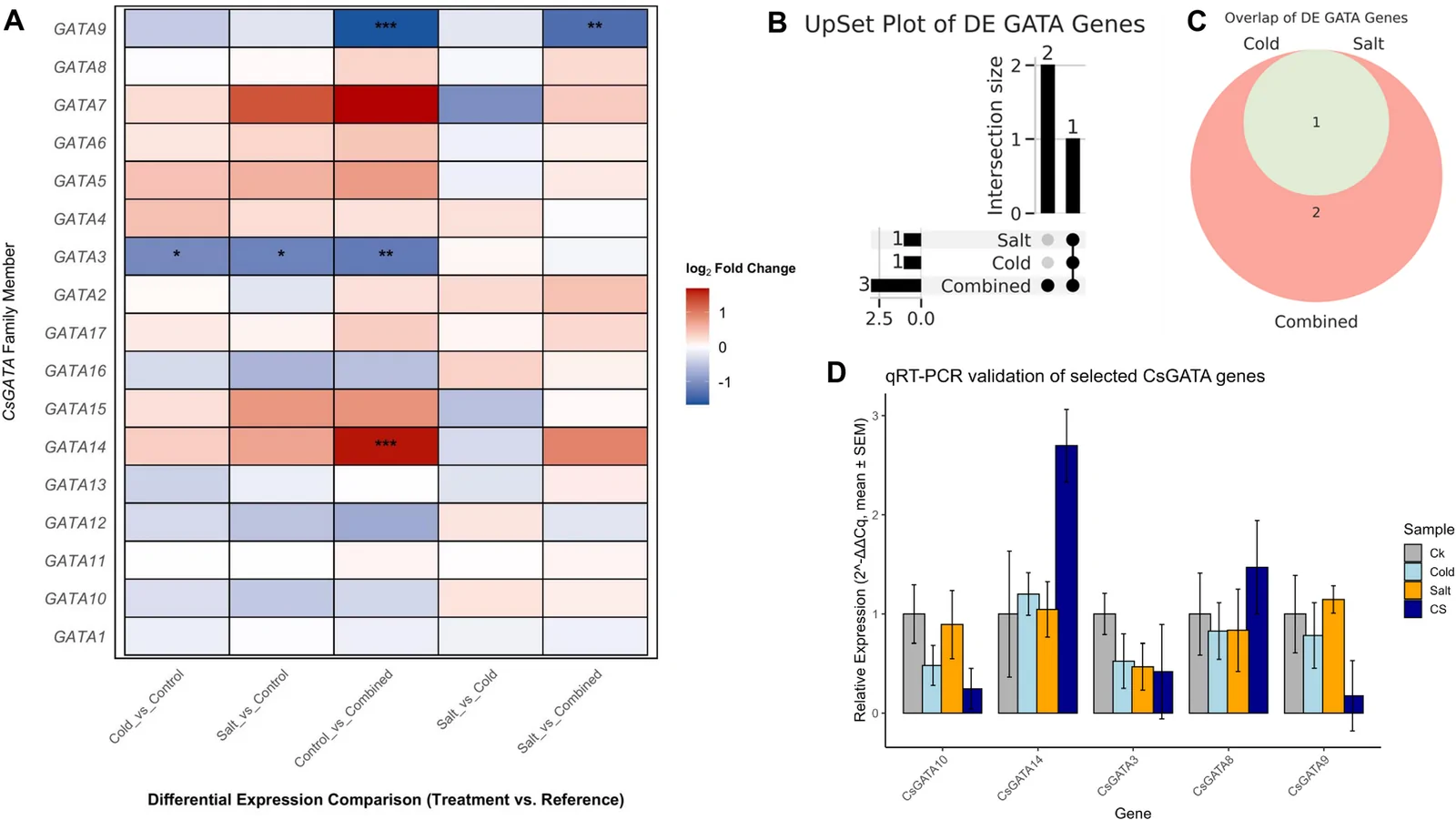
Differential expression profiles of *CsGATA* genes in response to abiotic stresses. **A** Heatmap depicting |log_2_FC| expression changes of *CsGATA* family members under cold, salt, and combined salt-cold stresses relative to control conditions. Pairwise comparisons between combined stress and single-stress treatments are reported separately in Supplementary Table S6. **B** UpSet plot illustrating the intersections and unique sets of differentially expressed (DE) *GATA* genes across salt, cold, and combined treatments. **C** Venn diagram showing the overlap of DE *GATA* genes among cold (one gene), salt (one gene), and combined (three genes) stress conditions. **D** qRT-PCR validation of relative expression levels for selected *CsGATA* genes under control (CK), salt, cold, and combined salt-cold (CS) treatments

Under salt stress (200 mM NaCl), *CsGATA3* was the sole DEG, exhibiting consistent and statistically significant downregulation (log_2_FC = −1.14; q < 0.05). Several other genes, including *CsGATA14*, *CsGATA15*, and *CsGATA16*, displayed statistically significant but modest changes (|log_2_FC|< 1). Similarly, cold stress (low-temperature) elicited a limited response, with only *CsGATA3* being significantly downregulated (log_2_FC = −1.08, q < 0.05). All other *CsGATA* members maintained stable expression levels (q > 0.05). Heatmap visualizations confirmed *CsGATA3* as the primary cold-responsive gene (Fig. 6A).

In contrast, the combined cold and salt stress treatment (synergistic stress) elicited stronger and more complex transcriptional changes. Three genes were significantly differentially expressed: *CsGATA3* (downregulated, log_2_FC = −1.23, q < 0.05), *CsGATA9* (downregulated, log_2_FC = −1.62, q < 0.05), and *CsGATA14* (upregulated, log_2_FC = + 1.62, q < 0.05). Several other genes, including *CsGATA5*, *CsGATA12,* and *CsGATA15*, showed moderate but statistically significant changes (|log_2_FC|< 1). The observed overlap of *CsGATA3* regulation across cold, salt, and combined conditions established it as a potential broad abiotic stress responder (Fig. 6B and C).

Pairwise comparisons revealed additional stress-specific signatures. Between salt and combined stress, *CsGATA9* was significantly more repressed in the combined treatment (log_2_FC = −1.37, q < 0.05). Between cold and combined stress, *CsGATA9* was induced (log_2_FC = + 1.16, q < 0.05), whereas *CsGATA14* was repressed (log_2_FC = −1.26, q < 0.05).

### Validation of RNA-seq expression profiles by qRT-PCR

**Fig. 7:**
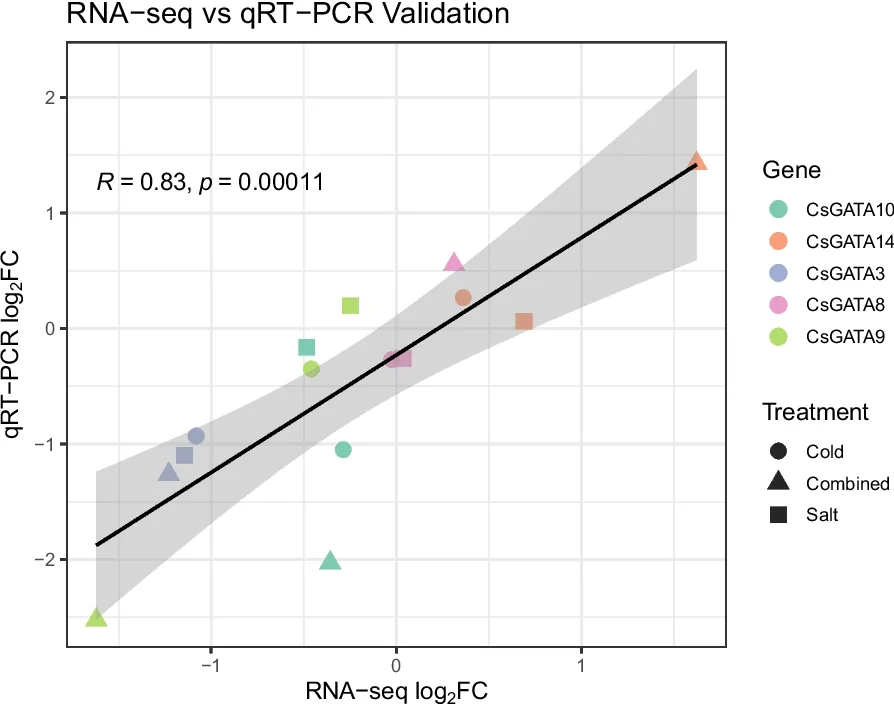
Validation of RNA-seq expression data by quantitative real-time PCR (qRT-PCR). Scatter plot showing the correlation between RNA-seq |log_2_FC| fold-change (treatment vs. control) and qRT-PCR |log_2_FC| (treatment vs. control calibrator) for five stress-responsive *CsGATA* genes (*CsGATA3*, *CsGATA8*, *CsGATA9*, *CsGATA10*, and *CsGATA14*) across three treatment conditions (cold stress, salt stress, and combined stress; *n* = 15 paired observations)

Five representative *CsGATA* genes, including *CsGATA3*, *CsGATA8*, *CsGATA9*, *CsGATA10*, and *CsGATA14* were selected to validate the reliability of the transcriptome data analysis under salt, cold, and combined stress conditions using qRT-PCR. The qRT-PCR expression patterns were generally consistent with the RNA-seq results (Fig. 6D). *CsGATA3* expression decreased to 0.45–0.52-fold under all stress treatments relative to control, consistent with transcriptome results. *CsGATA9* decreased to 0.17-fold under combined stress but remained stable under single stresses. *CsGATA14* was strongly induced under combined stress (2.71-fold), while *CsGATA8* and *CsGATA10* showed moderate responses (< 1.5-fold) that matched transcriptome trends. Pearson correlation analysis between RNA-seq |log_2_FC| values and qRT-PCR relative expression data demonstrated a strong positive correlation (*r* = 0.834, *p* < 0.001, *n* = 15), supporting the accuracy and reproducibility of the transcriptomic analysis (Fig. 7).

### Co-expression of CsGATA TFs with cell wall biosynthesis genes

**Fig. 8:**
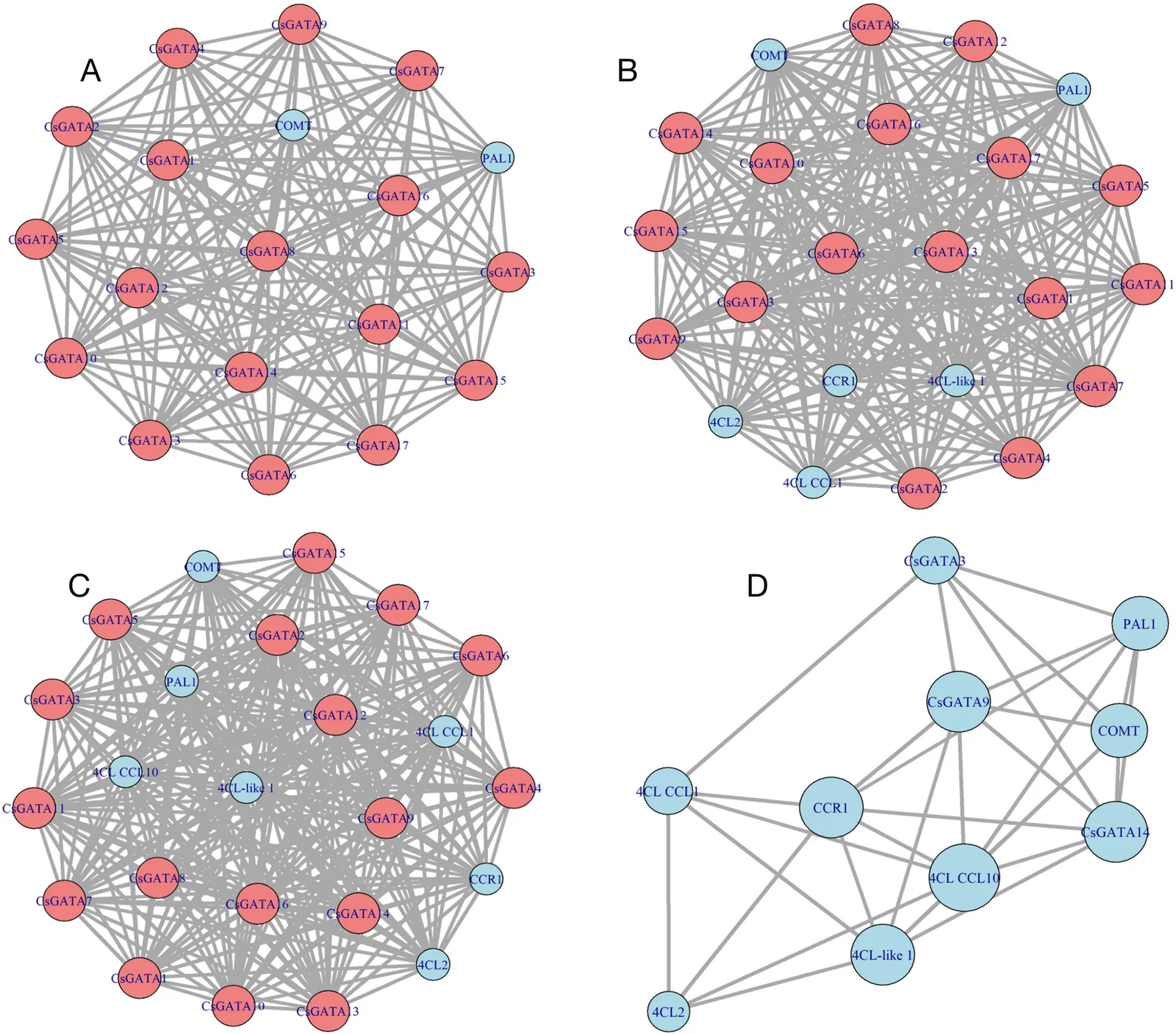
Co-expression networks between GATA transcription factors and phenylpropanoid pathway genes under abiotic stress conditions. **A** Co-expression network under cold stress (0 mM NaCl, 4 °C). **B** Co-expression network under salt stress (200 mM NaCl, 25 °C). **C** Co-expression network under combined cold and salt stress (200 mM NaCl, 4 °C). **D** Overall hub co-expression network displaying the top-ranked hub genes across all conditions based on degree centrality, including *4CL CCL10* (Degree = 8), *CCR1*, *4CL-like 1*, *CsGATA9*, and *CsGATA14* (Degree = 7 each)

To explore potential co-expression relationships between GATA TFs and secondary cell wall biosynthesis genes under abiotic stress, we constructed condition-specific and overall hub co-expression networks using Pearson correlation analysis. For each condition, networks were built by integrating all 17 *CsGATA* genes from the respective transcriptome data with phenylpropanoid pathway genes identified in the corresponding co-expression datasets (Fig. 8).

The overall hub genes co-expression network across all conditions (Fig. 8D) revealed a highly interconnected module. Hub gene analysis based on degree centrality identified *4CL CCL10* as the top hub (Degree = 8), followed by *CCR1, 4CL-like 1*, *CsGATA9*, and *CsGATA14* (all with Degree = 7) (Table 4). Importantly, the three stress-responsive *CsGATA* genes showed strong network connectivity: *CsGATA9* and *CsGATA14* (Degree = 7 each) and *CsGATA3* (Degree = 5). These *GATAs* exhibited robust positive correlations with key phenylpropanoid pathway genes, including *PAL1*, *COMT*, *CCR1*, and *4CL* family members.

**Table 4:** Top hub genes in the overall co-expression network between *CsGATAs* and phenylpropanoid/cell wall biosynthesis genes

Gene	Degree	Betweenness	Avg. Correlation
*4CL CCL10*	8	2.00	0.900
*CCR1*	7	4.17	0.793
*4CL-like 1*	7	2.00	0.826
*CsGATA9*	7	0.45	0.858
*CsGATA14*	7	0.45	0.800
*PAL1*	6	2.20	0.714
*COMT*	6	0.45	0.705
*4CL CCL1*	5	1.45	0.600
*CsGATA3*	5	0.83	0.613
*4CL2*	4	0.00	0.513

Condition-specific networks demonstrated dynamic rewiring depending on the stress type (Fig. 8A-C). The cold stress network (Fig. 8A) showed broad interconnectivity among *CsGATA* members but limited co-expression with phenylpropanoid pathway genes, with only *COMT* and *PAL1* meeting the correlation threshold (*r* ≥ 0.75). The salt stress network (Fig. 8B) revealed expanded pathway gene associations, with *CsGATA* members co-expressed with *PAL1*, *COMT*, *CCR1*, *4CL-like 1*, *4CL CCL1*, and *4CL2*. The combined stress network (Fig. 8C) displayed the most extensive associations, with particularly strong connectivity between *CsGATA9*, *CsGATA14*, and key lignin biosynthesis genes including *4CL CCL10*, *4CL-like 1*, *CCR1*, and *PAL1*, consistent with their identification as hub genes in the overall network (Fig. 8D). These patterns indicate stronger co-expression associations between GATA TFs and lignin biosynthesis genes under synergistic cold + salt stress, but they do not by themselves demonstrate direct regulatory relationships.

## Discussion

Transcriptional regulation is crucial for plant adaptation to environmental stress, especially during seed germination, when early developmental processes are strongly influenced by environmental conditions [1]. Although GATA TFs have been extensively studied in model plants [11], their genome-wide organization and stress-responsive behavior remain largely unexplored in industrial fiber crops. This study provides the first comprehensive characterization of the GATA TF family in *C. sativa* L. and integrates evolutionary, structural, transcriptomic, and co-expression analyses to elucidate their potential roles in abiotic stress responses during early germination.

Seventeen *CsGATA* genes were identified, a family size comparable to that reported in *A. thaliana* and *O. sativa* L. [11]. Instead of large-scale whole-genome duplication, tandem duplication events appear to have driven localized expansion, particularly in Clusters I and III. Such localized duplications are common among TF families and frequently contribute to functional diversification [53]. Phylogenetic and synteny analyses revealed both conserved and lineage-specific evolutionary patterns. Notably, the stress-responsive members (*CsGATA3*, *CsGATA9*, and *CsGATA14*) clustered within the largest clade (Cluster I), which also showed conserved synteny with *A. thaliana* and *O. sativa* L. *GATA* genes. This suggests that stress-responsive regulation in hemp is primarily mediated by evolutionarily conserved GATA lineages.

Consistent with *A. thaliana* and *O. sativa* L. [50], CsGATA proteins showed significant diversity in gene structure, physicochemical properties, and alternative splicing patterns. The high isoform diversity observed in genes such as *CsGATA2* and *CsGATA10* indicates that post-transcriptional regulation may enhance functional plasticity under stress conditions. Alternative splicing is increasingly recognized as a mechanism that enables plants to rapidly adjust TF activity under fluctuating environmental conditions, especially during stress-sensitive developmental phases [54].

All CsGATA proteins contained the conserved ZnF_GATA domain and were predicted to localize predominantly to the nucleus, consistent with their role as transcriptional regulators. Negative GRAVY values across the family further support their hydrophilic nature and compatibility with dynamic protein-DNA and protein–protein interaction. Collectively, these structural features indicate that *CsGATA* proteins possess the molecular attributes required for responsive transcriptional regulation during early development. Promoter analysis revealed enrichment of hormone and stress-responsive *cis*-elements (ABRE, MBS, and LTR), supporting the observed transcriptional responsiveness of several *CsGATA* genes. The presence of multiple ABREs in several promoters aligns with the known role of ABA signaling in seed germination and stress responses [55]. This heterogeneity aligns with the concept of functional partitioning within TF families, where individual members respond to distinct combinations of environmental and hormonal signals. In the context of seed germination, such diversification may enable fine-tuned regulation of growth, energy allocation, and stress tolerance during a critical physiological transition [56].

Transcriptomic profiling under cold, salt, and combined stress revealed that *CsGATA* genes exhibit highly specific and condition-dependent expression patterns under abiotic stress. Among all family members, *CsGATA3* was consistently downregulated across all conditions. This broad responsiveness suggests that *CsGATA3* may function as a stress-responsive transcriptional modulator whose downregulation is associated with growth restraint or transcriptional reprogramming under unfavorable physiological conditions. In contrast, *CsGATA9* and *CsGATA14* showed specific responses to combined stress. These patterns are consistent with the concept of functional partitioning within TF families, allowing plants to fine-tune responses to complex environmental conditions encountered during germination. To further examine the downstream regulatory roles of these stress-responsive *GATAs*, we performed condition-specific co-expression network analyses focusing on their association with phenylpropanoid and cell wall biosynthesis genes. The three stress-responsive *CsGATAs* (*CsGATA3*, *CsGATA9*, and *CsGATA14*) exhibited high network connectivity across conditions, with particularly strong associations to key lignin pathway genes (*PAL1*, *COMT*, *4CL* family members, and *CCR1*). These findings suggest that *CsGATAs* are associated with transcriptional responses linked to secondary cell wall remodeling under stress. However, the correlation network does not establish direct regulatory control over lignin biosynthesis genes. Similar regulatory links between transcription factors and cell wall defense genes have been reported in cotton under *Verticillium* wilt stress [57].

Seed germination represents a crucial physiological reference point in the plant life cycle, particularly for crops such as hemp that are often sown under suboptimal environmental conditions. Cold stress during imbibition can impair membrane fluidity and enzymatic activity [58], while salinity imposes osmotic and ionic constraints that disrupt cellular homeostasis [59]. The co-occurrence of these stresses can therefore exert compounded physiological pressure on germinating seeds. In this study, the focus on combined cold and salt stress addresses a biologically relevant scenario encountered during early-season hemp cultivation. The identification of *CsGATA* genes that respond specifically to combined stress conditions underscores the importance of regulatory mechanisms that integrate multiple environmental signals. Such integration is crucial for coordinating growth suppression, stress tolerance, and recovery during early establishment. Although this study does not establish direct causal roles for *CsGATA* genes, the integration of expression profiling with evolutionary and promoter analyses and co-expression analyses provides a strong foundation for functional inference. The consistent repression of *CsGATA3* across stress conditions, coupled with the stress-specific behavior of *CsGATA9* and *CsGATA14*, identifies these genes as promising candidates for future functional validation. Therefore, the potential involvement of *CsGATA* genes in coordinating stress responses with metabolic and developmental processes warrants further investigation.

## Conclusion

This study provides the first comprehensive genomic and transcriptomic characterization of the GATA TF family in *C. sativa* L. Seventeen *CsGATA* genes showing significant structural diversity and regulatory complexity were identified. Phylogenetic and synteny analyses revealed both conserved and lineage-specific evolutionary patterns, suggesting functional stability alongside adaptive diversification within the hemp genome. Transcriptomic analyses under cold, salt, and combined stress conditions identified *CsGATA3*, *CsGATA9*, and *CsGATA14* as key stress-responsive genes during early seed germination. Co-expression analysis across treatment conditions revealed that *CsGATA14* and *CsGATA9* emerged as highly connected hub genes within the overall correlation network linking GATA TFs with phenylpropanoid and secondary cell wall biosynthesis genes, including *CsCCR-1*, *CsCOMT* paralogs, and *Cs4CL* paralogs. Direct experimental validation of these regulatory relationships remains to be explored; these findings nonetheless identify *CsGATA14* and *CsGATA9* as promising candidates for future functional studies and molecular breeding strategies aimed at improving stress resilience and fiber quality in hemp.

## Supplementary Information

Supplementary Material 1: Figure S1. Dose-dependent effects of NaCl concentration and temperature on *C. sativa* L. seed germination and water uptake. (A) Cumulative germination curves over seven days after sowing for seeds treated with increasing NaCl concentrations (0, 100, 200, 300, 400, and 500 mM) at 25 °C (salt stress) and 4 °C (combined stress). (B) Final germination percentage at day 7 across all NaCl concentrations at both temperatures. Different lowercase letters above bars indicate statistically significant differences among treatments within each temperature regime (one-way ANOVA followed by Tukey’s HSD test, *p* < 0.05). (C) Seed water uptake kinetics expressed as percentage of dry seed weight over 48 h post-imbibition (hpi) across all NaCl concentrations at 25 °C and 4 °C.

Supplementary Material 2: Table S2. Summary of raw and clean sequencing read statistics for all RNA-seq libraries. For each of the 12 libraries (three biological replicates per treatment condition), the table reports total raw reads, raw base yield (Gb), raw read quality metrics (Q20%, Q30%, and N%), raw GC content (%), and the corresponding values after quality filtering with fastp (v0.23.1), including clean reads, clean base yield, clean Q20%, clean Q30%, clean N%, clean GC content (%), and the proportion of valid clean reads retained (Valid%). Conditions represented: cold stress (0 mM NaCl, 4 °C), combined stress (200 mM NaCl, 4 °C), salt stress (200 mM NaCl, 25 °C), and control (0 mM NaCl, 25 °C).

Supplementary Material 3: Table S3. HISAT2 alignment statistics for all RNA-seq libraries. Read mapping rates to the *C. sativa* L. reference genome (CS10; NCBI BioProject PRJNA557030) for each of the 12 libraries across four treatment conditions. The table reports total reads, total mapped reads (%), multiply mapped reads (%), and uniquely mapped reads (%) per replicate. All libraries achieved alignment rates exceeding 91%.

Supplementary Material 4: Table S4. Gene-specific primers used for qRT-PCR validation. Forward and reverse primer sequences for the five *CsGATA* genes selected for qRT-PCR validation (*CsGATA3*, *CsGATA8*, *CsGATA9*, *CsGATA10*, and *CsGATA14*) and the internal reference gene *CsActin*. Primers were designed using Primer3 software.

Supplementary Material 5: Table S5. NCBI Conserved Domain Database (CDD) search results for CsGATA proteins. Results of RPSBLAST-based domain annotation for all 17 CsGATA protein sequences against the CDD (NCBI). For each query, the table reports the hit type (specific or non-specific), PSSM-ID, domain start and end positions (From, To), E-value, bitscore, accession number, domain short name (ZnF_GATA), completeness status, and superfamily classification.

Supplementary Material 6: Table S6. Differential expression analysis of all 17 *CsGATA* genes across six pairwise stress comparisons. DESeq2-derived expression statistics for each *CsGATA* gene in six pairwise comparisons: (i) Control vs. Salt (200 mM NaCl, 25 °C), (ii) Control vs. Combined (200 mM NaCl, 4 °C), (iii) Control vs. Cold (0 mM NaCl, 4 °C), (iv) Salt vs. Combined, (v) Salt vs. Cold, and (vi) Combined vs. Cold.

## Data Availability

The Cannabis sativa reference genome (CS10) used in this study is publicly available under NCBI BioProject accession PRJNA557030. The RNA-seq raw sequencing data generated in this study has been submitted to the NCBI Sequence Read Archive (SRA) and will be publicly available upon acceptance of this manuscript. All other data supporting the findings of this study are included in the article and its supplementary materials.
