# Supplementary material for: Whole-genome analysis reveals the tandem duplication of Cannabis sativa L. GATA and their stress-responsive expression during seed germination
Source: BMC Plant Biol. 2026 Jun 29;26:1595. doi: 10.1186/s12870-026-09308-w (PMC13584446; doi:10.1186/s12870-026-09308-w)
Supplement: Supplementary file 1 [file 12870_2026_9308_MOESM1_ESM.pdf]

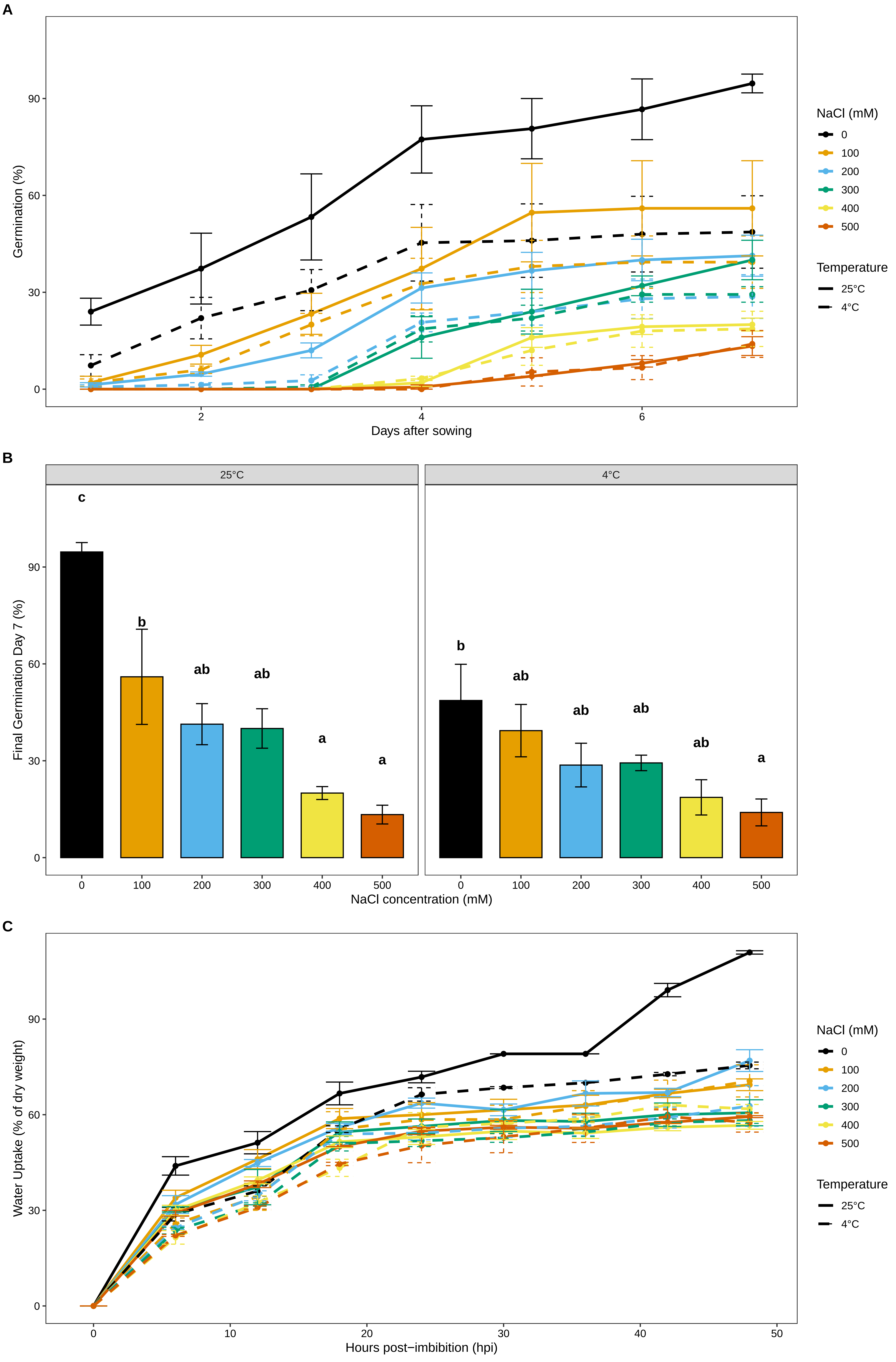

A
Germination (%)
Days after sowing
B
25°C
4°C
Final Germination Day 7 (%)
NaCl concentration (mM)
c
b
ab
ab
a
a
b
ab
ab
ab
ab
a
C
Water Uptake (% of dry weight)
Hours post−imbibition (hpi)
NaCl (mM)
0
100
200
300
400
500
Temperature
25°C
4°C
